# A Biostimulant Based on Silicon Chelates Enhances Growth and Modulates Physiological Responses of In-Vitro-Derived Strawberry Plants to In Vivo Conditions

**DOI:** 10.3390/plants12244193

**Published:** 2023-12-18

**Authors:** Elena Ambros, Olga Kotsupiy, Evgeniya Karpova, Ulyana Panova, Alexander Chernonosov, Elena Trofimova, Boris Goldenberg

**Affiliations:** 1 Central Siberian Botanical Garden, Siberian Branch of Russian Academy of Sciences, 101 Zolotodolinskaya Str., Novosibirsk 630090, Russia; 2 Institute of Chemical Biology and Fundamental Medicine, Siberian Branch of Russian Academy of Sciences, 8 Akad. Lavrentiev Ave., Novosibirsk 630090, Russia; 3 Institute of Solid State Chemistry and Mechanochemistry, Siberian Branch of Russian Academy of Sciences, 18 Kutateladze Str., Novosibirsk 630128, Russia; 4 Synchrotron Radiation Facility Siberian Circular Photon Source, Boreskov Institute of Catalysis, Siberian Branch of Russian Academy of Sciences, 1 Nikolsky Ave., Koltsovo 630559, Russia

**Keywords:** strawberry, silicon-chelate-based biostimulant, silicon accumulation, plant growth, cuticle, hydrogen peroxide, antioxidant defense, abscisic acid, in vivo conditions

## Abstract

The purpose was to assess the effects of a biostimulant based on silicon chelates in terms of alleviation of the impact of in vivo conditions on strawberry (*Fragaria* × *ananassa* cv. ‘Solnechnaya polyanka’) in-vitro-derived plants. As a source of silicon chelates, a mechanocomposite (MC) obtained through mechanochemical processing of rice husks and green tea was used. Root treatment of plants with 0.3 g L^−1^ of MC dissolved in tap water was performed at 2 weeks after planting. Control plants were watered with tap water. The greatest shoot height, number of roots per plant, root length, number of stolons per plant, daughter ramets per stolon, relative water content, cuticle thickness, and root and shoot biomasses were achieved with the MC supplementation. The improved parameters were associated with a higher silicon content of roots and shoots of the MC-treated plants. Leaf concentrations of hydrogen peroxide and abscisic acid were reduced by the MC. This effect was accompanied by enhanced activity of superoxide dismutase and catalase. The phenolic profile showed upregulation of *p*-hydroxybenzoic acid, vanillic acid, gallic acid, syringic acid, and ellagic acid derivative 2, while kaempferol rutinoside and catechins were downregulated. Thus, silicon chelates improve growth and trigger the physiological processes that enhance free-radical-scavenging activity in strawberry plants in vivo.

## 1. Introduction

Strawberry (*Fragaria* × *ananassa* (Duchesne ex Weston) Duchesne ex Rozier) is one of the most cultivated berries in the world. Due to their rich content of biologically active compounds, strawberry fruits are present in the diet of many people. At the same time, the crop, due to its superficial root system, large leaf area, and high water content of the fruits, is extremely sensitive to growth conditions during the cultivation process. The use of environmentally friendly approaches, such as biostimulants, can help sustainable strawberry agricultural practices. Biostimulants reduce the need for plant synthetic chemicals (fertilizers and pesticides) while contributing to the maintenance of an ideal quality and quantity of crop output [1,2].

At low concentrations, biostimulants trigger a number of physiological processes in a plant that enhance growth and yield and mitigate stress-induced limitations [3]. Plant biostimulants based on biogenic silicon (Si), which is absorbed by plants 2–10 times better than salts, are of great interest [4,5]. Silicon is not an essential element for plants, but its presence in different habitats has been shown to help increase plant resistance to negative environmental factors [6,7,8]. In many cases, exogenous Si treatments improve plant growth and increase productivity, thus enhancing adaptive reactions associated with the activity of the plant antioxidant system, accumulation of metabolites, changes in phytohormonal status, rate of photosynthesis, and nutrient elements’ uptake [4,9,10,11,12,13,14,15,16]. The beneficial effects of Si are attributed to the metabolic transformations caused by a modifying impact on sorption properties of the cell wall (where it accumulates in the form of amorphous silica) and by the formation of complexes with various organic compounds [17]. Nonetheless, the mechanisms and trend of Si absorption are different among plant species [18]. The foregoing is the dominant motive for further studies on the effect of Si on strawberry plants.

A promising source of Si-containing biostimulants is renewable plant raw materials obtained using a solid-phase mechanochemical method (without organic solvents) involving biogenic Si in chelate form. The method is also attracting much attention as a step toward green chemistry by reducing the use of hazardous chemicals. Because of such processing, plant raw materials and reagents remain in a stable solid form, which completely prevents their oxidation and loss of biological activity [19,20]. In addition, the presence of a wide range of plant wastes worldwide, including those containing Si, determines the demand for “green chemistry” as a component of organic farming for growing plants [21]. On the other hand, investigations of the behavior and interactions of silicon chelates in growing plants, the mechanism of their influence, and a variety of the effects during application in agriculture are still at a rudimentary stage.

Our previous studies about the effect of silicon chelates on in-vitro-derived strawberry plants have revealed improved growth and a contribution to changes in physiological characteristics. The physiological role of silicon chelates is thought to decrease contents of hydrogen peroxide and abscisic acid (ABA) and to promote activities of enzymatic antioxidants in the leaves, thereby mediating growth and the development of strawberry plants both under in vitro and ex vitro conditions [22,23]. These results lead us to the hypothesis that silicon chelates can similarly modulate the adaptability of strawberry microclones in vivo. A sustainable increase in stress tolerance of the microclones when transferred to in vivo conditions is important for creating an integrated industrial technological scheme to provide the basis for raising the yield of healthy strawberry planting material.

The leaf chlorophyll content is one of the main parameters indicating the physiological state of a plant [24]. This characteristic is often determined for agriculture monitoring and for evaluation of effects of various growth conditions or stimulants [25,26]. The concentration of photosynthetic pigments reflects plant adaptability and can point out a stressor in the environment [27,28,29].

Plant propagation starting from an explant in vitro to plant growth in the field is associated with sequential changes in the environment and growth conditions. Stress tolerance during all stages is a crucial factor for boosting productivity. The drastic alteration of the surroundings during the transfer of plantlets from in vitro to ex vitro conditions can cause an increase in the stress level and injuries of tissues. Oxidative damage can be triggered by overproduction of reactive oxygen species (ROS), represented predominantly by the superoxide anion (O_2_^−^), hydrogen peroxide (H_2_O_2_), the hydroxyl radical (•OH), and singlet oxygen (^1^O_2_), which cause the oxidation of cell compounds and suppression of plant growth and development. To protect cellular membranes and organelles, plants launch the antioxidant system that consists of both enzymatic and nonenzymatic mechanisms of detoxification. The nonenzymatic antioxidant system includes water-soluble (ascorbic acid and glutathione) and lipid-soluble antioxidants (α-tocopherol and β-carotene) [30]. Another essential part of the plant antioxidant system is phenolic compounds. Due to their aromatic rings and free hydroxyl groups, flavonoids and phenolic acids act as scavengers of ROS [31]. Superoxide dismutase (SOD), catalase (CAT), and peroxidase (PX) are the main enzymatic antioxidants in plants. They catalyze conversion of ROS via enzymatic defense reactions [32], and an increase in their activity is often registered in response to abiotic stress [33,34].

Plant hormones are involved in the regulation of the metabolic response to stress and are capable of inducing sequences of adaptive reactions. They govern plant growth in a wide range of environments [35,36]. Recently, the key role of ABA in plant responses to environmental stressors was documented [37]. ABA is generally maintained at low levels under non-stressful conditions, and its levels can rise significantly in response to environmental stressors and during seed maturation [38]. Effects of ABA on photosynthesis and ROS metabolism have been documented, too [39,40]. In addition, ABA is involved in the regulation of a plant’s water status [41] (through osmotic-stress-induced stomatal closure) as well as salt, drought, and cold tolerance [38]. The essential plant hormones cytokinins and auxins also participate in the stress response by maintaining plant growth and development under normal and stressful conditions [36].

Currently, more and more data are accumulating regarding Si’s ability to affect plant–environment interactions and to modify the response of the plant defense system [42]. In terms of stress tolerance, Si is regarded as a “multitalented” quasi-essential element. The manipulation of silicon sources to boost plant metabolic activity is starting a trend for sustainable horticulture [43]. Therefore, the present study was focused on assessing the effect of silicon chelates on the acclimation of strawberry plants to in vivo conditions and identifying changes in the growth, development, antioxidant defense system (enzymatic and nonenzymatic antioxidants), contents of photosynthetic pigments, and levels of phytohormones associated with the accumulation of silicon in shoots and roots of plants. An in-depth understanding of the influence of biogenic Si on plant growth and development will contribute to effective regulation of the crop fertilization system, which guarantees a consistently high yield of agricultural plants.

## 2. Results

### 2.1. The Si Content of In-Vitro-Derived Strawberry Plants

**Figure 1:**
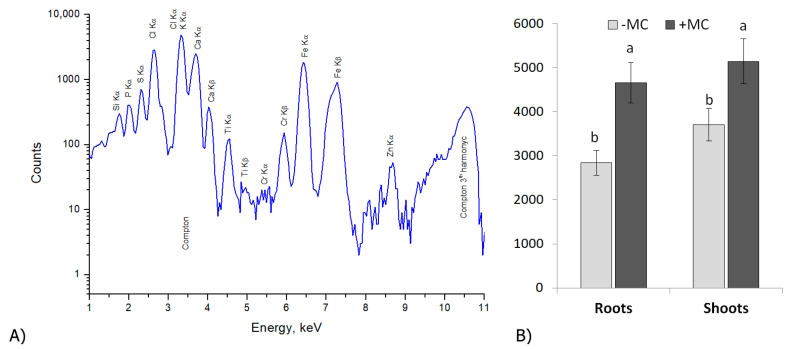
Typical X-ray fluorescence analysis with synchrotron radiation (SR-XRF) spectra of roots of in-vitro-derived strawberry plants (**A**). The silicon content of the roots and shoots in strawberry plants (cv. ‘Solnechnaya polyanka’) grown without the MC (−MC) or with MC treatment (+MC) in vivo (**B**). The data are means ± standard error. Different lowercase letters above the bars indicate significant differences (*p* < 0.05) between (−MC) and (+MC) treatment groups according to Duncan’s multiple-range test. Abbreviations: MC, the mechanocomposite based on silicon chelates; −MC, the mechanocomposite-free group; +MC, mechanocomposite treatment.

Si mainly accumulated in the shoots of the plants, not only in the group of plants supplemented with a mechanocomposite (MC; a source of silicon chelates) but also in the untreated (−MC) group (Figure 1). The Si content in shoots exceeded the Si content of plant roots by 1.1- and 1.3-fold in the MC-treated and untreated plants. The Si content of the strawberry plants grown without MC treatment (−MC) was lower than that of the MC-supplied plants (+MC). Significant elevation of the Si content was found both in the roots and shoots of the plants. The Si accumulation rose 1.6-fold in roots and 1.4-fold in shoots under the action of the (+MC) treatment compared with the (−MC) group (*p* < 0.05).

### 2.2. Growth Status and Vegetative Productivity of In-Vitro-Derived Strawberry Plants

**Figure 2:**
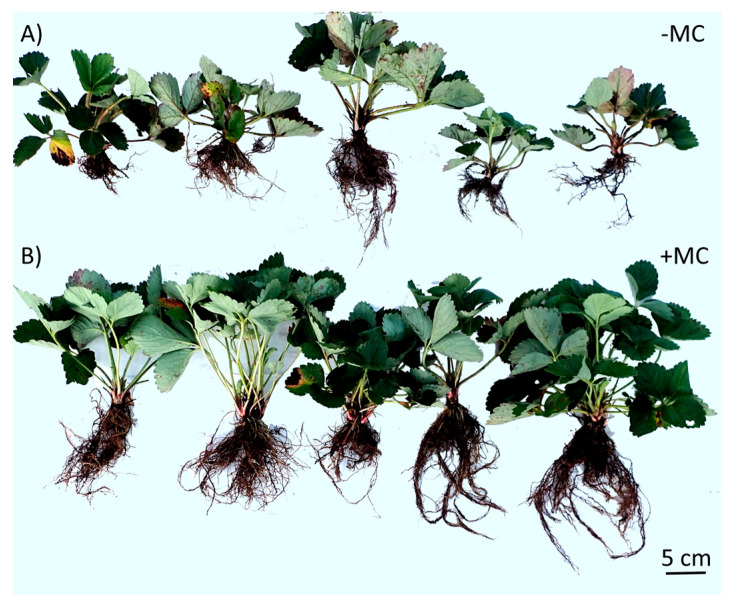
The phenotype of in-vitro-derived strawberry plants (cv. ‘Solnechnaya polyanka’) grown without the (−MC) (**A**) or with (+MC) treatment (**B**) in vivo. Abbreviations: MC, the mechanocomposite based on silicon chelates; −MC, the mechanocomposite-free group; +MC, mechanocomposite treatment.

The growth and development of the roots and above-ground parts of strawberry plants were significantly affected by the MC treatment (Figure 2B). Differences were identified for the root number, root length, and fresh and dry weights of the roots. Mean responses are shown in Table 1. Analysis of variance (ANOVA) indicated that root length in the (+MC) group was greater in comparison to the (−MC) group by 1.2-fold (*p* < 0.05); the root number was greater by 1.5-fold (*p* < 0.05). Root fresh weight and root dry weight were greater by 2.1-fold (*p* < 0.05) and 2.1-fold (*p* < 0.01), respectively.

A well-developed root system supported the best growth of above-ground parts of strawberry plants (Table 1). For instance, relative to the control (−MC), shoot height in the group with (+MC) was greater by 1.3-fold (*p* < 0.01), the number of leaves was greater by 1.4-fold (*p* < 0.05), and the fresh and dry weights of shoots were greater by 1.9 and 1.6-fold (*p* < 0.05), respectively.

**Figure 3:**
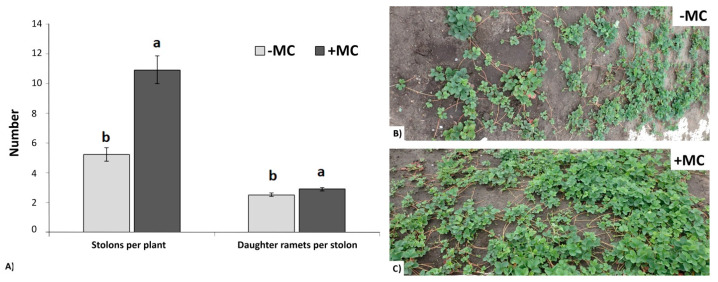
Vegetative productivity of in-vitro-derived strawberry plants (cv. ‘Solnechnaya polyanka’) grown without the MC (−MC) or (+MC) treatment in vivo. Formation of stolons and daughter ramets in the (−MC) group (**A**,**B**) and in the (+MC) group (**A**,**C**). The data are means ± standard error. Different lowercase letters above the bars indicate significant differences (*p* < 0.05) between (−MC) and (+MC) treatment groups according to Duncan’s multiple-range test. Abbreviations: MC, the mechanocomposite based on silicon chelates; −MC, mechanocomposite-free group; +MC, mechanocomposite treatment.

The pronounced effect of MC treatment was observed in stolons and daughter ramet formation (Figure 3). For instance, in the group with (+MC), the number of stolons per mother plant was greater by 2.1-fold (*p* < 0.05). In turn, the yield of daughter ramets per plant amounted to 50 compared to the group without MC, which produced an average of up to 7 ramets per plant (Figure 3A,C).

### 2.3. Physiological Characteristics of In-Vitro-Derived Strawberry Plants

Changes in essential physiological parameters of the plants are presented in Table 2 and Table 3. The relative water content (RWC) of the leaves of the plants was found to be significantly higher with (+MC) treatment (by 1.2-fold) (*p* < 0.05) in comparison to the (−MC) group. There was no noticeable difference in photosynthetic pigment concentrations between the (+MC) treatment group and the (−MC) group. In contrast, a significantly higher chlorophyll a/b ratio (*p* < 0.05) in the leaves of the (+MC) group was observed. In the presence of the MC, the chlorophyll a/b ratio was 1.2-fold higher compared with the control group.

The accumulation of H_2_O_2_ in the leaves was lower by 2.2-fold in the presence of the MC. Moreover, the plants grown with the MC exhibited significantly (*p* < 0.05) higher activities of the antioxidant enzymes SOD and CAT. SOD activity was greater by 1.1-fold, and CAT activity was greater by 3.5-fold (*p* < 0.05). POD activity was slightly higher compared with the (−MC) group (by 1.1-fold).

The quantification of phytohormones in the plant leaves showed that the ABA level was significantly lower in the (+MC) group (2.2-fold less than in the (−MC) (*p* < 0.05)). Differences in the indoleacetic acid (IAA) level were weaker. This phytohormone’s content grew (by 1.4-fold) during (+MC) treatment. The *trans*-zeatin (t-Z) level did not undergo a significant shift during the MC treatment.

The differences in the phenolic profile between the groups concerned mostly quantitative levels. The total phenolic concentration was higher by 14% compared to the control, and yet the impact on certain compounds was more significant. The most responsive to the treatment were hydroxybenzoic acids (Table 3). *p*-Hydroxybenzoic acid, vanillic acid, and syringic acid showed 1.67-, 1.44-, and 1.3-fold (*p* < 0.05) elevation of their levels in the (+MC) group. The concentration of gallic acid was only slightly higher, and the total hydroxybenzoic acid content was greater by 1.42-fold during the MC treatment.

The majority of hydroxycinnamic acids (neochlorogenic, *p*-coumaric, and ferulic acids) were present in the leaves at low concentrations and did not undergo the effect of the MC. In contrast, caffeic acid showed a substantial level, and it did not undergo significant elevation in the MC treatment group, either. Thus, a significant concentration shift of the total hydroxycinnamic acid content was not revealed during the MC treatment, which was similar to the total concentrations of ellagic acid and its derivatives. Only one compound from the latter group, ellagic acid derivative 2, exhibited substantial (1.57-fold) upregulation comparable to the compounds showing the greatest increase: *p*-hydroxybenzoic and vanillic acids. Similarly to hydroxycinnamic acids and ellagic acid derivatives, flavonol glycosides did not show a significant difference in the total concentration between the plant groups. Meanwhile, concentrations of quercetin glycosides were slightly higher, and the concentration of kaempferol rutinoside was significantly lower in the +MC group. Catechin concentration was slightly lower in the MC’s presence.

### 2.4. Cuticle Thickness in In-Vitro-Derived Strawberry Plants

**Figure 4:**
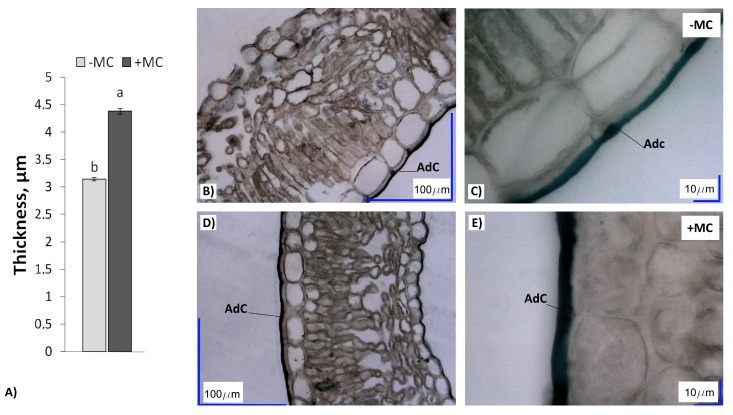
Cuticle thickness (**A**) and Sudan black B-stained transverse sections of leaves of in-vitro-derived strawberry plants (cv. ‘Solnechnaya polyanka’) grown without the MC (−MC) (**B**,**C**) or with the MC treatment (+MC) (**D**,**E**) in vivo. The data are means ± standard error. Different lowercase letters above the bars indicate significant differences (*p* < 0.05) between (−MC) and (+MC) treatment groups according to Duncan’s multiple-range test. Abbreviations: MC, the mechanocomposite based on silicon chelates; −MC, the mechanocomposite-free group; +MC, mechanocomposite treatment; AdC, adaxial cuticle.

The cuticle of strawberry leaves in the MC-supplied plants (+MC) was significantly thicker than that of plants grown without MC treatment (−MC) (Figure 4). This parameter in the leaves of (+MC) plants was higher by 1.4-fold compared with the (−MC) group (*p* < 0.05).

## 3. Discussion

Silicon accumulation has been found to be higher in monocotyledonous plants [44]. Most dicotyledons are unable to absorb large amounts of Si from soil. The difference in Si accumulation among species has been attributed to differences in the Si uptake ability of roots [45]. Thus, biochemical treatment of plant roots to enhance their ability to absorb Si and subsequently transfer Si to shoots can help plants overcome a wide range of stressors and improve metabolism [18]. A few studies present conflicting data on the absorption of Si by strawberry plants. There are several articles in which strawberry is classified as a plant that accumulates Si (up to 3% of dry weight) [46,47,48], whereas the work of M. J. Hodson et al. [49] suggests that strawberry does not absorb Si well. In our study, the elemental analysis confirmed the transfer of Si from the soil to the roots and Si accumulation in the strawberry shoots. Moreover, the strawberry plants absorbed Si from the soil in amounts that benefited the plant. The MC treatment significantly affected the growth and biomass of both roots and shoots of the strawberry plants. This finding is consistent with the results of other studies, indicating Si-mediated enhancement of root and shoot development and also showing active accumulation of Si mainly in organs of cereal and vegetable crops [50]. It is believed that under the influence of Si, the architecture of the cell wall changes and becomes stronger because this element accumulates in rhizodermis cells of the root cap and exudates of root hairs, leading to a total increase in the root surface adsorption [17,51]. After the transport from roots to shoots, Si is accumulated as amorphous silica in cell walls of shoot endodermis, mainly in the transpiration area of shoots, and becomes immobile without relocating anywhere [45,52,53]. These deposits of Si protect plants from multiple abiotic and biotic stresses.

Besides vegetative growth, the MC treatment increased the quantity of ramets from the mother plant. Supposedly, Si can be transported as a nutrient from mother plants to daughter plants. For instance, Y. Li et al. [54] have reported that the foliar spray and root drench of mother plants significantly promote Si accumulation in daughter plants, whereas spraying the runner plants does not increase the Si content of the mother plants. Such changes are undoubtedly useful in the production of strawberry planting material.

Additionally, Si treatment of plants results in the formation a cuticle–Si double layer [55], which helps the plants to hold more water. In our work, the increase in cuticle thickness and in the RWC of the strawberry leaves after the MC treatment confirms other authors’ data about the effect of Si on plant water transport processes, including leaf water loss, vessel water transport, and root water uptake [56,57]. In leaves, the deposition of silica phytolith on the cuticle and stomata has been shown to decrease the transpiration loss of water under water deficit stress [58]. It is probable that the increase in the RWC in our experiment was related to both a higher water absorption capacity of plant roots under the influence of the MC and a low rate of its loss by leaves. The increase in the root system’s biomass, leading to larger root surface area, proves this supposition. In turn, the low leaf water loss is confirmed by greater silicon accumulation in the shoots of the MC-treated strawberry plants.

Moreover, our results align with the reports describing Si as a trigger that induces physiological mechanisms allowing a strawberry plant to improve resistance to biotic stress by means of defensive compounds and enzymes [59,60]. The main stressors for strawberry plants in the field are suboptimal temperatures, a water deficit or excess, extreme solar radiation, and biotic threats of different natures (bacteria, fungi, or insects). Physiological parameters in our work highlighted a significant effect of the silicon-chelate-based MC on phytohormone and antioxidant systems of strawberry plants. Moreover, the results allow us to correlate some physiological parameters of the plants growing in the field with those of in vitro plantlets [22]. H_2_O_2_ levels and activities of antioxidant enzymes vary strongly throughout the propagation cycle [22], and their concentrations are under a strong influence of the MC. In the absence of the MC, a spike in the H_2_O_2_ level (4.23 and 123.27 µmol g^−1^ during in vitro and in vivo growth, respectively) was registered. Nevertheless, in the +MC treatment group, the increase in H_2_O_2_ during in vivo growth was not so considerable (56.08 µmol g^−1^). At the same time, CAT activity without the MC was modest (10.8 and 8.33 (U H_2_O_2_)(g FW]^−1^ min^−1^) during in vitro multiplication and in vivo growth, respectively), but it was almost 3-fold higher in the MC treatment group (10.4 and 29.51 (U H_2_O_2_)(g FW)^−1^ min^−1^]. SOD and POD activities rose manyfold from the in vitro to in vivo stage. Meanwhile, during the in vivo growth with MC treatment, the increase in SOD activity was not so pronounced (98.28 vs. 103.74 U (g FW)^−1^ in (−MC) and (+MC) groups), and POD activity did not change significantly compared to (−MC) conditions (2.78 vs. 3.13 U (g FW)^−1^) [22].

The level of chlorophyll a substantially went up in the leaves of the plants in the field (1.31 and 1.38 mg g^−1^ FW in the −MC and +MC groups) compared to the in vitro plantlets (0.30 and 0.22 mg g^−1^ FW) [22]. In contrast, the chlorophyll b level did not undergo pronounced changes (0.39 and 0.30 vs. 0.33 and 0.30 mg g^−1^ FW). Obviously, the MC has no significant effect on the chlorophyll content. The carotenoid level also rose throughout plant development (0.14 and 0.10 vs. 0.44 and 0.46 mg g^−1^ FW). Meanwhile, the influence of the MC on this parameter was not substantial either, especially at the in vivo stage.

Our results point out the key role of ABA in the response to Si accumulation. N. Kumar et al. [61] have demonstrated the regulation of the genes responsible for Si accumulation by plant hormones. One of the stress hormones involved in the regulation of Si transport in plants is ABA [62]. Our results are in agreement with this pattern of a stress response, where stress hormones govern Si accumulation after an environmental trigger. Therefore, the lower ABA level in the leaves of the strawberry plants of the MC treatment group may be a consequence of the involvement in Si accumulation. The participation of auxins and cytokinins in Si effects has been poorly investigated [63]. Under in vivo conditions here, these hormones did not exhibit their correlation with the Si effect, yet their potential participation in the implementation of Si’s effects may be of interest for our further research. These plant hormones support normal growth and development under stressful conditions. Meanwhile, the precise mechanism of auxin- and cytokinin-mediated stress tolerance is yet to be discovered [36].

Another part of the plant’s response to Si application may be a concentration shift of certain phenolic compounds [60]. ABA is reported to take part in the transformation of phenolic metabolism through upregulation or downregulation of the genes related to phenylpropanoid and flavonoid metabolism [64]. G. Hao et al. [65] have demonstrated an enhancement of the activity of phenylalanine ammonia-lyase and chalcone synthase, which are crucial for flavonoid accumulation, in response to the upregulation of ABA. Therefore, the simultaneous decline in the ABA level and the increase in the phenolic compound concentration in the strawberry plants in the MC treatment group may indicate that silicon is a trigger of phenolic compound accumulation [59,60]. This decline in the ABA level may be caused by a reduction in stress-induced signals in MC-treated plants, which possess improved mechanical parameters, and, consequently, a reduced risk of biotic threats and an elevated water content.

In the last few years, data have been accumulating regarding the effects of Si compounds on plants, including, among them, induction of a concentration shift of phenolic compounds as a part of a plant’s defense system. Meanwhile, these data are still fragmentary and contradictory. The main reasons for their variation are the differences in the plant genotype, environment, age, physiological state, Si form, concentration in the treatment, and stressor type. I. Vega et al. [66] have revealed a decrease in lipid peroxidation and intensive lignin accumulation induced by Si treatment, mainly with a subsequent reduction in concentrations of caffeoylquinic acid and flavone glycosides in barley under aluminum stress. In an experiment with *Passiflora incarnata*, Si application induced upregulation of total polyphenols and vitexin [67]. A. Carneiro-Carvalho et al. [68] have registered elevation of levels of the major phenolic compounds having antifungal activity in Si-treated chestnut plants up to 2–4-fold compared to untreated ones. The enhancement of production of phenolic compounds was also registered by G. Shahnaz et al. [69] during mitigation using silicon of the negative impact of Al stress on *Borago officinalis*.

The growth of many plants in the field has been shown to be accompanied by high levels of quercetin glycosides, which are effective antioxidants and defensive compounds [70,71]. R. Hajiboland et al. [72] have observed a Si-mediated increase in the leaf concentration of quercetin at three developmental stages of strawberry plants (vegetative, flowering, and fruiting stages). A considerable increase in the concentration of quercetin glycoside rutin (more than 20-fold) induced by Si, with powdery mildew severity reduced, has been revealed in *Rosa hybrida* plants [73]. The strawberry plants in our study similarly contained substantial concentrations of quercetin glycosides, but they were not significantly higher in the +MC group. Consequently, our results do not indicate participation of quercetin glycosides in the response to Si application. In contrast to quercetin glycosides, substantial downregulation of kaempferol rutinoside in the strawberry leaves was revealed in the MC treatment group. Accumulation of kaempferol and/or its glycoside in the cell wall and thickened cuticle of MC-treated plants could be behind this decrease. The essential role of kaempferol glycosides in cuticle formation of some plant species has been demonstrated before [74,75,76].

Our results are in agreement with R. Hajiboland et al.’s findings [72] about significant upregulation of phenolic acids under the influence of silicon. They showed consistently higher leaf concentrations of gallic, caffeic, and chlorogenic acids in +Si plants at all three developmental stages. Benzoic acids (gallic, *p*-hydroxybenzoic, vanillic, and syringic acids) were the main class of phenolic compounds that ensured the elevation of the total level of phenolic compounds in the strawberry leaves in the field under the influence of Si. Similar phenolic acid upregulations in other plants have also been reported [60,72,73]. These have been associated with the strengthening of plant protection against bacteria, fungi, and herbivores [73,77,78,79,80]. It is noteworthy that the decrease in the concentrations of catechins and ellagic acid derivatives in the strawberry leaves treated with Si may be caused by their polymerization and the formation of compounds having a strong defensive effect against various biotic threats [81,82].

In spite of some discrepancies between the literature’s data on the responses to Si application, the upregulation of phenolic compounds, including phenolic acids and flavonols, is often recorded. Our findings confirm the involvement of phenolic compounds in the effect of Si on antioxidant status and biotic resistance of strawberry plants and show alterations of the phenolic profile in strawberry leaves under the influence of Si. The downregulation of some compounds under the influence of Si, as observed in our study and some previous papers [66,72,83], can perhaps be attributed to their accumulation in cell-wall-bound fractions [72] and the formation of high-molecular-weight polyphenols [84].

Thus, the results indicate a positive effect of MC treatment on the metabolism and anatomy of cells and tissues of strawberry plants. In the future, to expand the scope of the experiment, we would like to focus on the effect of the MC on soil–nutrient relations. Elemental analysis of the strawberry plants during MC treatment in our greenhouse uncovered a significant increase in the levels of micronutrients (Cu, Zn, Fe, and Mn) (unpublished data). The involvement of the studied biochemical and anatomical alterations in the overall effect on plant nutrition is interesting primarily.

## 4. Materials and Methods

### 4.1. Preparation and Characterization of Silicon Chelates

As a source of silicon chelates, an MC obtained through mechanochemical processing of plant raw materials was used [20]. The MC was produced from rice husks (*Oryza sativa*, cv. ‘Liman’, Krasnodar Krai, Russia) and green tea (*Camellia sinensis* L.) (Krasnodar Krai, Russia) through mechanical activation in a water-cooled flow activator (roller mill RM-20, manufactured at the Institute of Solid State Chemistry and Mechanochemistry, SB RAS, Novosibirsk, Russia). The MC was found to contain 1.4% ± 0.2% of catechins (mean ± SE), 16.7% ± 0.9% of ash, and 15.2% ± 0.7% of silica. In the MC, the mass ratio of rice husks to green tea was 10:1.

### 4.2. Experimental Design

The field experiment was conducted on the experimental plot of the Central Siberian Botanical Garden (CSBG), SB RAS, Novosibirsk, Russia (geographical coordinates 54°49′9.87″ N and 83°6′6.95″ E), during May–August 2022. Dimensions of the experimental plot were 4.0 × 4.0 m. Mechanical properties of the soil in this area are as follows: medium loamy with a large amount of coarse dust fractions, structureless, with poor air and water permeability, rapid settlement and compaction after processing, and a propensity for swimming and crusting. Its pH is slightly acidic at 6.3–6.9. The soil layer from 0 to 20 cm contains 2–4% humus, and at a depth of 50–60 cm, no more than 0.8%. The total natural reserves of nutrients are low; therefore, humus was introduced into the holes during planting. 

In-vitro-derived strawberry plants (*F.* × *ananassa* cv. ‘Solnechnaya polyanka’) were grown in plastic pots containing 250 cm^3^ of a soil substrate from a mixture of peat with perlite, humus, sand, and a coco substrate (1:1:0.25:0.25, *v*/*v*/*v*/*v*) and were used for planting under field conditions. Single-fruiting cv. ‘Solnechnaya polyanka’ is characterized by stable high productivity and is recommended for cultivation under Western Siberia conditions. 

There were two accounting plots of 4.0 × 1.20 m for the control group and treatment group on the experimental plot. The plants were planted in rows according to a 0.40 × 0.35 m scheme. The row area was 0.4 m × 1.2 m, and 10 plants per row were planted. The experiment was conducted in four biological replicates for each treatment type. One factor was analyzed statistically: the MC application.

Plants were treated by root watering with 0.3 g L^−1^ of MC (the +MC group) dissolved in tap water (2.7 L per plant) at 2 weeks after planting. The control group of plants was watered with tap water (−MC). The experiment consisted of three treatments during the growing season at regular intervals (3 weeks). In between, when the soils dried out, the plants were moistened with water. The plants’ above-ground parts were sampled 1 week after the last treatment with the MC to assess the growth and physiological characteristics and Si accumulation. 

### 4.3. Analysis of Growth Characteristics

Growth was measured in terms of shoot height (cm), the number of leaves and roots per plant, root length (cm), the number of stolons per plant, the number of daughter ramets per stolon, fresh and dry weights of shoots and roots (g), and leaf blade area (cm^2^).

The dry weight of the plant material was determined by drying it three times to constant weight at 75 °C for 2 h.

The area of plant leaf blades was calculated using the SIAMS Photolab software with the additional module SIAMS MesoPlant Ekaterinburg, Russia).

### 4.4. Determination of the RWC

This parameter of leaf tissues is used to assess the water status of plants, and it is expressed by: RWC = [(FW − DW)/(TW − DW)] × 100, where FW, DW, and TW are fresh, dry, and turgid weights, respectively, of a plant [85]. To determine the turgid weight, leaf sections (10 mm) were placed in distilled water at 23 ± 2 °C under light for 24 h. The dry mass was measured after drying the leaves at 75 °C for 48 h. For this analysis, the leaves of 20 randomly selected plants from each treatment group were used. The samples for pigment concentrations, analysis of hydrogen peroxide and enzymatic antioxidants, and phytohormone quantification were prepared from combined and homogenized fresh leaves of 20 randomly selected plants from each treatment group. Similarly prepared samples of air-dried leaves were used for analysis of phenolic compounds.

### 4.5. Determination of Pigment Concentrations

Fresh leaf tissues (10 mg) were ground in 96% ethanol (1000 µL). The homogenate was centrifuged using Eppendorf Centrifuge 5430 R (Hamburg, Germany) at 8000 rpm for 10 min. After that, the supernatant’s volume was brought to 3 mL with 96% ethanol. The optical density (D) of the solutions was measured immediately at wavelengths of 470, 664, and 649 nm on a UNICO 2100 UV spectrophotometer (Princeton, New Jersey, NJ, USA). The samples containing free 96% ethanol served as a control. The pigment concentration (C) was calculated according to H.K. Lichtenthaler and C. Buschmann [86] using the following formulas:

C_a_ = (13.36 × A_664_ − 5.19 × A_649_);

C_b_ = (27.43 × A_649_ − 8.12 × A_664_);

C_c_ = (1000 × A_470_ − 2.13 × C_a_ − 97.64 × C_b_)/209,

where C_a_, C_b_, and C_c_ are concentrations of chlorophyll a, chlorophyll b, and carotenoids (mg mL^−1^) and A_664_, A_649_, and A_470_ are the extract’s absorbance at 664, 649, and 470 nm.

The concentrations of pigments (C_P_) in leaf tissue were calculated and expressed in milligrams per gram of fresh weight (FW) (mg g^−1^) as follow: C_P_ = C × V/(1000 × n), where C is pigment concentration in the ethanol extract (mg mL^−1^); V is the volume of the extract (mL); and n is the fresh weight of the sample (g).

### 4.6. Analysis of Hydrogen Peroxide and Enzymatic Antioxidants

The endogenous hydrogen peroxide (H_2_O_2_) level and enzymatic activities were assayed according to previously published protocols [22]. The H_2_O_2_ content was measured based on the peroxide-mediated oxidation of Fe^2+^, followed by the reaction of Fe^3+^ with xylenol orange [87,88]. Absorbance of the Fe^3+^–xylenol orange complex was measured at 560 nm. A standard curve was obtained by diluting 3% H_2_O_2_. The data were expressed in micromoles of H_2_O_2_ per gram of FW (μmol (g FW)^–1^).

Catalase (EC 1.11.1.6) activity was determined according to the decomposition of H_2_O_2_ [89] and measured on the UNICO 2100 UV spectrophotometer (USA) by assessing a decrease in absorbance at 240 nm. The reaction mixture contained 50 mM of Na-K-phosphate buffer (pH 7.0) and 0.03 mL of enzyme extract. CAT activity was expressed in units of H_2_O_2_ decomposed per minute per gram of FW ((U H_2_O_2_)(g FW)^−1^min^−1^).

Superoxide dismutase (EC 1.15.1.1) activity determination involved ethylenediaminetetraacetic acid (EDTA), a 0.05% (*w*/*v*) nitro blue tetrazolium solution, a 0.025% (*w*/*v*) riboflavin solution, and 0.1 mL of the enzyme extract [90]. The reaction mixture was incubated for 15 min at 25 °C under light with an intensity of 40 μmol m^−2^ s^−1^ (Philips, Pila, Poland), and then absorbance was measured at 560 nm against a nonirradiated blank. Absorbance was expressed in units (U) per gram of FW (U (g FW)^–1^).

Peroxidase (EC 1.11.1.7) activity was measured at 365 nm as a decrease in quercetin concentration [91]. The POD reaction solution contained 0.1 mL of the enzyme extract, 0.2 M of acetate buffer (pH 4.5), 0.1 μM of quercetin, and 10 μM of H_2_O_2_. The reaction mixture contained 50 mM of Na-K-phosphate buffer (pH 7.8) and 0.24% (*w*/*v*) of added H_2_O_2_. The activity was expressed in units of quercetin oxidized per minute per gram of FW (U (g FW)^–1^ min^–1^).

### 4.7. Phytohormone Quantification

Quantification of IAA, ABA, and t-Z was performed using the method of Dobrev et al. [92]. Standards of phytohormones were purchased from Sigma-Aldrich: IAA (Sigma-Aldrich, Chemie GmbH, Buch, Switzerland), ABA (Sigma-Aldrich, Trading Co Ltd, Shanghai, China), and t-Z (Sigma-Aldrich, AG Industriestrasse, Karnataka, India). Internal standards indolepropionic acid (IPA), naphthylacetic acid (NAA), and 6-benzylaminopurine (BA) were acquired from Sigma-Aldrich, CHEMIE GmbH (Shanghai, China). Liquid chromatography/mass spectrometry (LC-MS)-grade acetonitrile was purchased from Biosolve (Dieuze, France). Water for LC-MS was obtained using a Milli-Q purification system from Millipore Corp. (Bedford, MA, USA). Gaseous nitrogen (ultrapure, 99.9%) was produced by means of a Genius NM32LA nitrogen generator (Peak Scientific Instruments Ltd., Scotland, UK). LC-MS-grade acetic acid was acquired from Sigma-Aldrich (St. Louis, MO, USA). LC coupled with tandem mass spectrometry (MS/MS) was performed at the Core Facility of Mass-Spectrometric Analysis of ICBFM SB RAS for quantification of IAA, ABA, and t-Z in an extract. For this analysis, 100 mg of FW of leaves (above-ground parts) of strawberry plants was homogenized in the mixture of methanol, water, and formic acid (15:4:1); internal standards (BA 50 ng and IPA and NAA 500 ng each) were added into the mixture. The homogenate was put into a 2 mL microcentrifuge tube, which was placed into a freezer (–20 °C) for extraction for 1 h. After that, the homogenate was spun in the centrifuge at 20,000× *g* at 4 °C for 20 min. The supernatant was transferred into a new 2 mL tube and evaporated to a volume less than 0.25 mL.

Chromatographic separation was performed on an Agilent 1200 HPLC system (Agilent Technologies, Santa Clara, CA, USA) outfitted with a Prontosil C-18 analytical column (Econova, Novosibirsk, Russia) with an Eclıpse XDB-C18 pre-column (Agilent Technologies, Santa Clara, CA, USA). MS detection was conducted on an Agilent 6410 QQQ mass spectrometer (Agilent Technologies, USA) equipped with an electrospray ionization (ESI) source. For IAA and t-Z quantification, detection was carried out in the positive multiple-reaction monitoring (MRM) mode, and for ABA detection, the negative MRM mode was applied.

In the negative mode, transition *m*/*z* 263.0 → 219.1 was employed for quantification of ABA (collision energy 14 eV, fragmentation voltage 130 V) and transition *m*/*z* 185.1 → 140.9 was selected for NAA (collision energy 5 eV, fragmentation voltage 130 V). In positive mode, the following transitions were utilized for quantification of analytes: *m*/*z* 220.1 → 136.1 for t-Z (collision energy 15 eV, fragmentation voltage 110 V), 226.1 → 91.1 for BA (collision energy 33 eV, fragmentation voltage 100 V), *m*/*z* 176.1 → 130.1 for IAA (collision energy 10 eV, fragmentation voltage 70 V), and 190.1 → 130.1 for IPA (collision energy 15 eV, fragmentation voltage 130 V). Typical MS conditions were spray voltage at 4000 V, turbo spray temperature of 300 °C, and nitrogen as a nebulizer gas at 40 psi and a 9.2 L min^−1^ flow rate. The duration of a single ion scan was set to 500 ms for t-Z, IAA, AA, and NAA and to 200 ms for BA and IPA. The temperature of the autosampler was adjusted to 6 °C.

For the chromatographic separation of the phytohormones and internal standards, gradient elution with 5 mM of acetic acid in water (eluent A) and 0.5 mM of acetic acid in acetonitrile (eluent B) was conducted. The run with the gradient was started with a solvent A–solvent B mixture at 95:5 (*v*/*v*) followed by a linear gradient to 50:50 (*v*/*v*) for the first 15 min, then to 10:90 (*v*/*v*) from minute 15 to minute 20, and this proportion was constant until minute 22; the mobile phase was returned to 95:5 from minute 22.0 to minute 22.1, and this proportion was constant up to minute 30. The flow rate was set to 0.3 mL·min^−1^. Calibration curves for the phytohormones’ quantification were built using several concentrations: 1, 5, 10, 30, 50, and 80 ng·mL^−1^. Data were processed using the Agilent Technologies Masshunter B03.01 [B2065] software.

### 4.8. High-Performance Liquid Chromatography (HPLC) Analysis of Phenolic Compounds

For this analysis, air-dried above-ground parts from three randomly selected plant samples were used. Dry weight concentrations in the samples were calculated using the gravimetric method. Then, the samples were ground up, weighed (0.2 g), and extracted three times with an ethanol–water mixture (70:30, *v*/*v*) in a water bath at 60–70 °C. HPLC analysis of the aqueous ethanol extracts was performed using an Agilent 1200 system with a diode array detector and ChemStation B04.01 SP1[647] software for data processing (Agilent Technologies, Santa Clara, CA, USA). The chromatographic separation was carried out on a Zorbax SB-C18 column (4.6 × 150 mm, 5 μm internal diameter) at 25 °C. The mobile phase consisted of MeOH (solvent A) and 0.1% orthophosphoric acid in water (solvent B).

The separation of compounds in the extracts was performed through gradient 1. The run via this gradient was started with an A–B mixture at 22:78 (*v*/*v*) followed by a linear gradient to 70:30 (*v*/*v*) for the first 30 min, and then to 100:0 (*v*/*v*) from minute 30 to minute 32. The mobile phase was returned to 22:78 (*v*/*v*) from minute 32 to minute 36.

To confirm glycoside composition, flavonoid aglycones were identified after acid hydrolysis of the extracts. The aqueous ethanol extracts of above-ground parts of *F.* × *ananassa* were hydrolyzed with 2N HCl for 1 h in a boiling water bath. The hydrolyzed extracts were separated via gradient 2. The run via this gradient was started with a solvent A–solvent B mixture at 50:50 (*v*/*v*) followed by a linear transition to 52:48 (*v*/*v*) for the first 15 min, then to 100:0 (*v*/*v*) from minute 15 to minute 17, with a return to 50:50 (*v*/*v*) from minute 17 to minute 20. The flow rate in both gradients was set to 1 mL·min^−1^. The sample injection volume was 10 μL. Monitoring of chromatograms was carried out via absorbance at 220, 255, 270, 290, 325, 340, 350, and 370 nm.

Phenolic compounds were quantified using the external standard method. Reference standards of gallic, *p*-hydroxybenzoic, syringic, neochlorogenic, *p*-coumaric, caffeic, and ellagic acids and of (+)-catechin, quercetin, kaempferol, kaempferol rutinoside (Sigma-Aldrich, St. Louis, MO, USA), epigallocatechin gallate (Teavigo, Gevelsberg, Germany), vanillic acid (Serva, Heidelberg, Germany), and ferulic acid (Koch-light Lab Ltd., Haverhill, Suffolk, UK) were used for building calibration curves in the concentration range of 10–100 μg mL^−1^. Ellagic acid derivatives and quercetin glycosides were quantified as ellagic acid and rutin, respectively.

Three biological and three technical replicates were processed in each assay.

### 4.9. X-ray Fluorescence Analysis of Si

Samples of roots and shoots of 20 plants from each treatment group were dried at 50 °C to a constant weight. Then, the material was ground and pressed into tablets weighing 20 mg. The Si content of the samples was determined through SR-XRF at the Siberian Synchrotron and Terahertz Radiation Center (SSTRC) at Budker Institute of Nuclear Physics, SB RAS, Novosibirsk, Russia (BINP SB RAS) at the 1st SR beamline of the VEPP-4M synchrotron [93]. Elemental analysis was carried out using monochromatic exciting synchrotron radiation at 3.53 keV photon energy. The characteristic X-rays emitted from the sample were detected using a Si-PIN diode X-ray detector (Amptek XR-100CR, Bedford, MA, USA; with 165 eV FWHM of MnKα, 500 μm Si crystal thickness, and 12.7 μm Be window) positioned at 45° from the normal of the sample surface. The beam size at the sample position was ~1 × 4 mm. For calculation of the Si content, a procedure of the external standard analysis using a standard sample (the Tr1 meadow herbal mix; CRM 8922 2007, produced at Vinogradov Institute of Geochemistry, SB RAS, Irkutsk, Russia) was applied. The concentrations of Si were expressed in ppm (μg g^−1^).

### 4.10. The Cuticle Thickness Measurement

For the analysis, the second leaf was taken from the apical bud of the shoot. Then, 5.0 × 5.0 mm sections were cut out from the middle part of the middle lobe of the trifoliate leaf, avoiding the midrib. The collected leaf materials were fixed in FAA (formalin: glacial acetic acid: 95% ethanol, 7:7:100 (*v*/*v*/*v*)) before storage in 70% ethanol. After that, the leaves were dehydrated using a graded ethanol series. Dehydrated leaf materials were sequentially passed through mixtures made of 95% ethanol/Technovit 7100 resin (Heraeus Kulzer, Germany) combinations (7:3, 15 h; 1:1, 15 h; 3:7, 15 h (*v*/*v*)) and then 100% Technovit for 15 h. The samples were then solidified in Technovit 7100 resin following the manufacturer’s protocol. Embedded samples were cut on an HM-325 rotary microtome (Microm, Germany) into sections of 7 μm thickness, placed onto slide glass, and dried on an electric slide warmer for 12 h. Dried slides were stained with 0.2% Sudan black B (Servicebio Technology Co., Ltd., Wuhan, China) for 60–90 s, rinsed with running water, and again dried by means of the electric slide warmer for more than 6 h to remove water. The stained slides were then mounted with Mowiol^®^ (Sigma-Aldrich, Taufkirchen, Germany) and examined under a VHX-5000 digital light microscope (Keyence, Osaka, Japan). For each type of sample, 300 morphometric measurements were carried out.

### 4.11. Statistical Analysis

For the data analyses, STATISTICA 10.0 software (Statsoft Inc., Tulsa, OK, USA) was used. The results of the number of roots per plant, root length, fresh and dry weights of shoots and roots, and leaf blade area were reported as mean ± standard errors (SE) of 20 individual plants. The results of shoot height, the number of leaves per plant, and the number of stolons per plant were expressed as mean ± standard errors (SE) of 40 individual plants. The results of the biochemical tests and analysis of Si were presented as mean ± SE of 9 (3 biological x 3 technical) replicates. The results were compared using Duncan’s multiple-range test to find significant differences among treatment groups. Differences between the means were considered statistically significant at *p* < 0.01 and *p* < 0.05.

## 5. Conclusions

The present results revealed that Si application has beneficial effects on in-vitro-derived strawberry plants during growth in the field. Silicon-chelate-mediated metabolic alterations in the strawberry plants resulted in improvement of protective defense mechanisms in the plants. These strategies involve a complex antioxidant defense system that is composed of antioxidant enzymes and low-molecular-weight compounds (ABA, benzoic acids, and ellagic acid derivative). The activation of the antioxidant defense system promoted growth of the strawberry plants and substantially enhanced their vegetative productivity due to an increase in biomass and in the number of ramets from a mother plant. Cuticle layer thickening in strawberry leaves is proposed as a potential mediator of the modulation of the water balance by silicon chelates. These findings may help produce commercial strawberries worldwide.

## Figures and Tables

**Table 1:** Effects of MC treatment on growth-related parameters of in-vitro-derived strawberry plants (cv. ‘Solnechnaya polyanka’) during in vivo acclimation.

Parameter	Treatment Type	
	(−MC) Group	(+MC) Group
root length (cm)	13.32 ± 0.75 b *	18.00 ± 1.27 a *
roots (number per plant)	13.30 ± 1.47 b *	20.35 ± 1.49 a *
root fresh weight (g)	3.18 ± 0.40 b *	6.52 ± 0.92 a *
root dry weight (g)	0.83 ± 0.08 b **	1.72 ± 0.16 a **
shoot height (cm)	10.88 ± 0.42 b **	14.26 ± 0.63 a **
leaves (number per plant)	10.17 ± 0.66 a	14.13 ± 1.16 a
leaf blade area (cm^2^)	39.57 ± 4.34 a	43.43 ± 3.00 a
shoot fresh weight (g)	14.18 ± 1.54 b *	26.30 ± 3.11 a *
shoot dry weight (g)	3.33 ± 0.25 b *	5.35 ± 0.54 a *

The data are means ± standard error. Different lowercase letters within a row indicate significant differences (* *p* < 0.05; ** *p* < 0.01) between (−MC) and (+MC) treatment groups according to Duncan’s multiple-range test. Abbreviations: MC, the mechanocomposite based on silicon chelates; −MC, the mechanocomposite-free group; +MC, mechanocomposite treatment.

**Table 2:** Effects of MC treatment on physiological parameters in leaves of in-vitro-derived strawberry plants (cv. ‘Solnechnaya polyanka’) during in vivo acclimation.

Parameter	Treatment Type	
	(−MC) Group	(+MC) Group
RWC (%)	55.92 ± 1.71 b	64.62 ± 2.40 a
chlorophyll a (mg g^−1^ FW)	1.31 ± 0.05 a	1.38 ± 0.04 a
chlorophyll b (mg g^−1^ FW)	0.33 ± 0.01 a	0.30 ± 0.01 a
total chlorophyll (mg g^−1^ FW)	1.64 ± 0.07 a	1.67 ± 0.05 a
chlorophyll a/b ratio	3.94 ± 0.07 b	4.66 ± 0.13 a
carotenoids (mg g^−1^ FW)	0.44 ± 0.02 a	0.46 ± 0.02 a
total chlorophyll/carotenoids	3.93 ± 0.31 a	3.66 ± 0.06 a
H_2_O_2_ (μmol (g FW)^–1)^	123.27 ± 24.19 a	56.08 ± 14.40 b
SOD (U (g FW)^–1^)	98.28 ± 1.21 b	103.74 ± 0.22 a
CAT ((U H_2_O_2_)(g FW)^−1^ min^−1^)	8.33 ± 2.85 b	29.51 ± 10.14 a
POD (U (g FW)^–1^ min^–1^)	2.78 ± 1.00 a	3.13 ± 0.07 a
IAA (ng g^−1^ FW)	6.60 ± 1.01 a	9.37 ± 0.83 a
ABA (ng g^−1^ FW)	3.57 ± 0.61 a	1.63 ± 0.10 b
t-Z (ng g^−1^ FW)	2.65 ± 0.49 a	2.06 ± 0.74 a

The data are means ± standard error. Different lowercase letters within a row indicate significant differences (*p* < 0.05) between (−MC) and (+MC) treatment groups according to Duncan’s multiple-range test. Abbreviations: MC, the mechanocomposite based on silicon chelates; −MC, the mechanocomposite-free group; +MC, mechanocomposite treatment; RWC, the relative water content; FW, fresh weight; H_2_O_2_, hydrogen peroxide; CAT, catalase; POD, peroxidase; SOD, superoxide dismutase; IAA, indoleacetic acid; ABA, abscisic acid; t-Z, trans-zeatin.

**Table 3:** Effects of MC treatment on nonenzymatic antioxidants in leaves of in-vitro-derived strawberry plants (cv. ‘Solnechnaya polyanka’) during in vivo acclimation.

Phenolic Compound, mg g−1 DW	t_R_, min	λ_max_, nm	Treatment Type	
			(−MC) Group	(+MC) Group
Hydroxybenzoic acids:				
gallic acid	2.4	216, 272	0.17 ± 0.01 a	0.18 ± 0.01 a
*p*-hydroxybenzoic acid	6.8	254	0.07 ± 0.00 b	0.12 ± 0.01 a
vanillic acid	9.4	260, 290	4.03 ± 0.18 b	5.82 ± 0.28 a
syringic acid	9.7	220, 270	0.51 ± 0.03 b	0.69 ± 0.02 a
Total			4.78 ± 0.21 b	6.81 ± 0.26 a
Hydroxycinnamic acids:				
neochlorogenic acid	3.8	240, 296 sh., 325	0.15 ± 0.01 a	0.17 ± 0.01 a
caffeic acid	8.7	218, 240, 298 sh., 324	1.19 ± 0.05 a	1.26 ± 0.06 a
*p*-coumaric acid	10.8	226, 310	0.11 ± 0.01 a	0.11 ± 0.05 a
ferulic acid	13.3	218, 236, 320	0.04 ± 0.00 a	0.04 ± 0.00 a
Total			1.49 ± 0.07 a	1.58 ± 0.07 a
Catechins:				
(+)-catechin	5.3	204, 230 sh., 280	0.55 ± 0.03 a	0.49 ± 0.02 a
epigallocatechin gallate	7.7	204, 230 sh., 280	0.09 ± 0.00 b	0.06 ± 0.00 a
Total			0.64 ± 0.04 a	0.55 ± 0.02 a
Ellagic acid and its derivatives:				
ellagic acid	17.7	254, 367	1.04 ± 0.05 a	1.13 ± 0.06 a
ellagic acid derivative 1	8.0	230, 262	1.00 ± 0.04 a	1.13 ± 0.06 a
ellagic acid derivative 2	11.2	230, 276	1.16 ± 0.05 b	1.82 ± 0.05 a
ellagic acid derivative 3	12.4	254, 362	0.74 ± 0.03 a	0.53 ± 0.02 b
ellagic acid derivative 4	16.3	254, 360	2.55 ± 0.14 a	2.26 ± 0.14 a
ellagic acid derivative 5	16.8	254, 360	0.75 ± 0.04 a	0.65 ± 0.04 a
Total			7.24 ± 0.35 a	7.52 ± 0.21 a
Flavonol glycosides:				
kaempferol rutinoside	19.2	265, 356	0.23 ± 0.01 a	0.13 ± 0.01 b
quercetin glycoside 1	13.8	270, 355	4.78 ± 0.27 a	5.48 ± 0.35 a
quercetin glycoside 2	14.0	270, 355	1.54 ± 0.07 a	1.61 ± 0.07 a
Total			6.55 ± 0.36 a	7.23 ± 0.41 a
Total phenolic compounds			20.70 ± 1.03 a	23.69 ± 0.81 a

The data are means ± standard error. Different lowercase letters within a row indicate significant differences (*p* < 0.05) between (−MC) and (+MC) treatment groups according to Duncan’s multiple-range test. Abbreviations: MC, the mechanocomposite based on silicon chelates; −MC, the mechanocomposite-free group; + MC, mechanocomposite treatment; sh., shoulder; t_R_, retention time; λ_max_, absorption maxima.

## Data Availability

Raw data from this study are available upon request from the corresponding author.
